# Chemical Composition, In Silico Investigations and Evaluation of Antifungal, Antibacterial, Insecticidal and Repellent Activities of *Eucalyptus camaldulensis* Dehn. Leaf Essential Oil from ALGERIA

**DOI:** 10.3390/plants13223229

**Published:** 2024-11-17

**Authors:** Ghozlane Barboucha, Noureddine Rahim, Houssem Boulebd, Amina Bramki, Anna Andolfi, Maria Michela Salvatore, Marco Masi

**Affiliations:** 1 Biotechnologies Laboratory, Higher National School of Biotechnology Taoufik Khaznadar, Nouveau Pôle Universitaire Ali Mendjeli, Constantine 25100, Algeria; g.barb@ensbiotech.edu.dz (G.B.); n.rahim@ensbiotech.edu.dz (N.R.); 2 Laboratory of Synthesis of Molecules with Biological Interest, University of Frères Mentouri Constantine 1, Constantine 25017, Algeria; boulebd.houssem@umc.edu.dz; 3 Laboratory of Bio Engineering, Higher National School of Biotechnology Taoufik Khaznadar, Nouveau Pôle Universitaire Ali Mendjeli, Constantine 25100, Algeria; a.bramki@ensbiotech.edu.dz; 4 Department of Chemical Sciences, University of Naples Federico II, 80126 Naples, Italy; andolfi@unina.it (A.A.); mariamichela.salvatore@unina.it (M.M.S.); 5 BAT Center–Interuniversity Center for Studies on Bioinspired Agro-Environmental Technology, University of Naples ‘Federico II’, Portici, 80055 Naples, Italy

**Keywords:** *Eucalyptus camaldulensis*, essential oil, antibacterial, antifungal, insecticidal, DFT calculations, molecular docking

## Abstract

This study investigated the phytochemical profile and evaluated the antimicrobial and insecticidal properties of *Eucalyptus camaldulensis* Dehn. essential oil (EC-EO) from Algeria, using in vitro and in silico approaches. The yield of EC-EO was 0.27%, with gas chromatography-mass spectrometry (GC-MS) revealing spathulenol (58.24%), cryptone (17.22%), and *o*-cymene (15.53%) as the major compounds. EC-EO exhibited notable antibacterial activity, particularly against *Salmonella typhimurium* (14 ± 1.00 mm) and *Staphylococcus aureus* (14.5 ± 0.50 mm). It also showed effective antifungal activity against *Penicillium* sp. (11.5 ± 0.49 mm), *Candida albicans* (11.2 ± 0.29 mm), and *Aspergillus fumigatus* (9.8 ± 0.27 mm). Insecticidal assays against *Tribolium castaneum* were conducted using contact toxicity, fumigation toxicity, and repellent activity methods. The median lethal concentration (LC50) for contact toxicity was 0.011 μL/insect after 72 h, while the fumigation test had an LC50 of 122.29 μL/L air. Repellent activity tests showed percentage repellency (PR) values exceeding 80% after 6 h. The molecular geometry and electronic properties of the main compounds were studied using density functional theory (DFT) calculations. In addition, the interaction mode and binding affinity of these molecules with three key enzymes involved in antimicrobial activity, DNA gyrase, dihydrofolate reductase (DHFR) and Tyrosyl-tRNA synthetase (TyrRS), were explored by molecular docking.

## 1. Introduction

Over the past several decades, the promotion of natural products for daily use has gained global momentum, with essential oils (EOs) emerging as some of the most widely utilized [1]. EOs are natural volatile secondary metabolites derived from aromatic plants, known for their distinctive fragrances and a wide range of bioactive properties [2]. Due to their potential health benefits and natural origins, these oils are applied in various fields, including the food industry, medicine, agriculture and cosmetics [3,4,5]. For example, EOs are being more frequently utilized in food preservation due to their ability to inhibit microbial growth, thereby extending shelf life and ensuring food safety [6]. Pharmaceutically, they show potential as natural treatments for infections, with research highlighting their effectiveness against multidrug-resistant bacteria, positioning them as valuable candidates in the fight against antibiotic resistance [7,8]. In the agricultural sector, their insecticidal properties enhance pest control strategies, contributing to more sustainable and environmentally friendly practices [9,10,11]. Due to their high value, the development of essential oils from plants and the evaluation of their activities are still in great demand.

Among the diverse range of EOs, *Eucalyptus* species have attracted significant attention for their rich phytochemical composition and remarkable biological activities. They show considerable potential for the prevention and treatment of viral infections, serve as powerful inhibitors of oxidative stress, and display effective antimicrobial activity against various pathogens [12,13]. Widely used around the world, *Eucalyptus* essential oils are recognized by the U.S. Food and Drug Administration (FDA) as safe and non-toxic, and the Council of Europe has approved their use as flavoring agents in foods [14]. Meanwhile, in Japan the extract of eucalyptus leaves appears on the List of Food Additives as an antioxidant [15]. Eucalyptus is an evergreen, non-native species in Algeria, currently cultivated extensively throughout the country [16]. The introduction of this genus to Algeria occurred in 1854, initiated by Ramel, with *Eucalyptus globulus* being the first species introduced [17]. Native to Australia, this genus belongs to the Myrtaceae family and comprises over 700 species, some of which have applications in traditional medicine for treating various medical conditions [18,19]. Additionally, certain *Eucalyptus* species are widely employed in modern medicine. For instance, *Eucalyptus camaldulensis* is used in formulations such as expectorants for treating lung diseases and coughs [20,21]. Furthermore, it exhibits antibacterial, antifungal, microbiostatic, antituberculosis, and disinfectant properties [22,23].

Despite its widespread use and biological activities, the chemical composition and bioactivity of *E. camaldulensis* EO from Algeria remain inadequately explored. Given the increasing demand for natural alternatives in pest and disease management, this study aims to investigate the chemical composition of *E. camaldulensis* leaf EO using gas chromatography-mass spectrometry (GC-MS), and evaluate its antifungal, antibacterial, insecticidal, and repellent properties against storage-grain insects. To the best of our knowledge, this study represents the first investigation into the insecticidal potential of *E. camaldulensis* EO from Algeria, providing critical data for the development of sustainable biopesticides.

## 2. Results and Discussion

### 2.1. Composition of E. camaldulensis Essential Oil

The *Eucalyptus camaldulensis* EO was yellow, with a yield of 0.27% (*w*/*w*). The constituents of the obtained EO are listed in Table 1. This yield is lower than those reported for *E. camaldulensis* leaves collected in Algeria and Morocco, which ranged from 0.42% to 0.75% [24,25,26], as well as other yields recorded in Mediterranean regions. For example, Barra et al. [27] reported a yield of 0.50% in Sardinia, Italy, and Jaradat et al. [28] found 1.1% in Jenin, Palestine. The highest yield of essential oil from *E. camaldulensis* was recorded in Taiwan, ranging from 2.3% to 3.0% (*w*/*w*) [29].

These variations in EO yields are likely due to different environmental factors. Age and sampling season may also affect EO yield [30].

**Figure 1:**
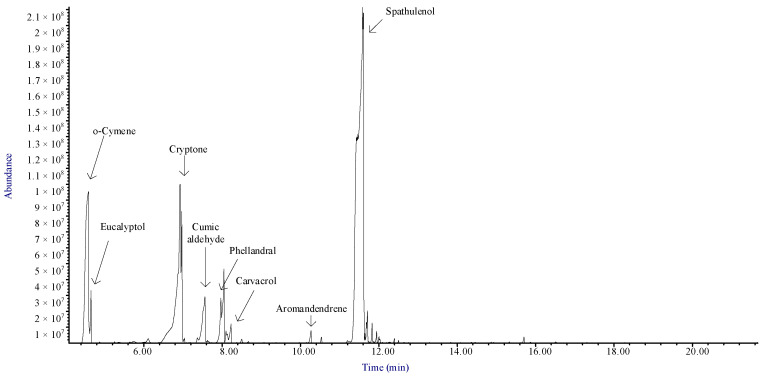
Annoted total ion current chromatogram (TICC) from GC-MS analysis of the essential oil extracted from *E. camaldulensis* leaves.

GC-MS analysis indicated that EC-EO is predominantly composed of sesquiterpenes, with spathulenol (sesquiterpene alcohol) as the main component at 58.24%. Monoterpenes represent the second most prevalent class, with cryptone (monoterpene ketone) at 17.22% and o-cymene (monoterpene hydrocarbon) at 15.53%. Other notable compounds identified include phellandral (monoterpene aldehyde) at 3.63%, cumic aldehyde (aromatic aldehyde) at 2.92%, and eucalyptol (monoterpene ether) at 1.22%. (Table 1 and Figure 1).

These findings align with those reported by Knezevic et al. [31] in Montenegro, where spathulenol (21.39%) and cryptone (12.15%) were also identified as dominant compounds, though in lower proportions. Similarly, Elgat et al. [32] noted that spathulenol constituted 20.84% of the oil, followed by p-cymene (15.16%), 1,8-cineole (12.01%), and sabinene (9.73%). Additionally, Achour et al. [26] showed a similar composition in northern Algeria (Tizi Ouzou), with spathulenol (22.05%) and cryptone (16.79%) as key components, along with a notable presence of p-cymene (22.50%). However, a study by Ez-Zriouli et al. [24] in Morocco reported a distinct profile for *E. camaldulensis* EO, identifying p-cymene (35.11%), γ-eudesmol (11.9%), and l-linalool (11.51%) as the major constituents.

Variations in the qualitative and quantitative composition of essential oils can result from several influencing factors, including regional topography, climate (temperature and humidity), soil characteristics, plant age, genotype, maturity stage, and the extraction methods applied [33,34,35,36].

### 2.2. Antimicrobial Activity

#### 2.2.1. Antibacterial Activity

The highlight test by disc diffusion technique demonstrated the effectiveness of *E. camaldulensis* EO compared to the positive control to inhibit the growth of all tested strains except *P. aeruginosa* (Table 2). The inhibition zones diameter ranged from 8.0 ± 0.3 to 14.5 ± 0.5 mm. The most substantial effect was observed against *S. typhimurium* and *S. aureus* with inhibitions zones diameters of 14.0 ± 1.0 mm and 14.5 ± 0.5 mm, respectively. However, *B. subtilis*, *E. coli* and *K. pneumoniae* were less sensitive to EO where the measured diameters were 11.0 ± 1.0 mm, 10.0 ± 0.4 mm and 8.0 ± 0.3 mm, respectively.

Our findings align with those reported in previous studies, where *E. camaldulensis* EO exhibited antibacterial activity against different bacterial strains. Notably, Ghaffar et al. [37] found inhibition zones ranging from 10 to 24 mm against *S. aureus*, *B. subtilis* and *E. coli*. In addition, Mehani and Segni [38] reported inhibition zone of 9.7 mm against E. coli. Which is comparable to our results, 6.3 mm against *P. aeruginosa*, 11.7 against *Enterobacter* sp. and 10 mm against *Proteus* sp. However, some discrepancies in the diameter of inhibition zones may be due to differences in experimental conditions, such as the concentration of EO, technique used, and incubation time.

The bioactive EO was found to be majorly composed of spathulenol, cryptone, and o-cymene, all of which are well documented for their antibacterial activities. According to Fernandes et al. [39], spathulenol inhibited the growth of *Cryptococcus neoformans* and *Enterococcus faecalis*. Its activity has been also reported against *S. aureus*, *S. epidermidis*, *P. aeruginosa*, *Enterobacter cloacae*, *K. pneumonia* and *E. coli* [40]. Also, Agnaniet et al. [41], reported that the antibacterial properties of *E. polybractea* EO would be enhanced by cryptone. Furthermore, o-cymene is a geometric isomer of p-cymene with ortho-substituted alkyl groups which have been proven to have an antibacterial potency against Gram-positive and Gram-negative bacteria [42]. Its chemical similarity to our molecule suggests it may have comparable antibacterial properties.

#### 2.2.2. Antifungal Activity

Antifungal activity of *E. camaldulensis* EO against four fungal strains exhibited moderate efficacy compared to the positive control (Table 3).

The most substantial inhibition zones were observed with *C. albicans* and *Penicillium* sp. measuring 11.16 ± 0.29 mm and 11.50 ± 0.49 mm in diameter respectively. Following these, *A. fumigatus* displayed an inhibition zone with a diameter of 9.83 ± 0.27 mm. Conversely, the least pronounced activity was observed with *A. niger* with an inhibition diameter of 7.0 ± 0.50 mm. Our results align with those of previous studies on *E. camaldulensis* EO. Hamdoon et al. [43] corroborated our results, reporting an inhibition zone of 8.0 ± 0.20 mm for *A. niger* and 22.0 ± 0.30 mm for *C. albicans*. However, Diriye et al. [44] observed larger inhibition zones, measuring 18 mm for *A. niger* and 30 mm for *C. albicans*. Similarly, Ghaffar et al. [37] reported an inhibition zone of 28 ± 0.83 mm against *A. niger* and 22 ± 0.82 mm against *Rhizopus solani*. These differences in inhibition diameters may be attributed to variations in the genetic makeup of the tested strains. Furthermore, differences in experimental conditions, such as the concentration or composition of the essential oil, could also account for the observed variations. The compounds present in EO play a crucial role in its antifungal activity. Spathulenol, which constitutes 58.24% of the total composition, is well recognized for its antifungal properties. According to Cazella et al. [45], EO from *Baccharis dracunculifolia*, predominantly composed of spathulenol, showed both fungistatic and fungicidal activities against *A. fumigatus*, *A. niger*, *A. versicolor*, *A. ochraceus*, *P. funiculosum*, *P. ochrochloron*, *P. verrucosum* and *Trichoderma viride*. Additionally, cryptone, present at 17.22%, has been reported to exhibit significant antifungal activity. Agnaniet et al. [41] found that various strains of *C. albicans* were sensitive to cryptone in the essential oils extracted from the bark of *Glossocalyx staudtii*. Furthermore, previous research has demonstrated that o-cymene, accounting for 15.53% of our EO, can inhibit the growth of *Botrytis cinerea*, *C. albicans*, *A. niger* and *A. flavus* [46,47,48]. This activity can be attributed to the hydrophobicity of EOs and their major component, which allows them to interact with the lipid membranes of fungal cells and mitochondria, thereby altering their structure and increasing their permeability. Consequently, this interaction may lead to ion leakage or the release of other cellular constituents [49].

### 2.3. Insecticidal Activity

#### 2.3.1. Contact Toxicity

The contact topical toxicity of *E. camaldulensis* EO against *T. castaneum* is shown in Table 4 and Table 5. The data showed that EO tested exhibited an interesting toxicity towards adults of *T. castaneum*. Moreover, in the treatment of EO against this insect, mortality was a concentration-dependent response (Table 4).

In fact, the mortality percentages increased with high concentrations of the EO. The mortality percentage values ranged from 30.0 to 100%. The highest mortality percentage was achieved at the concentration of 0.3 μL/insect (Mortality = 100%) after 72 h. A significant difference mortality was observed across different concentrations of EO and the time after treatment: at 24 h (F = 16.56, *p* < 0.05), 48 h (F = 16.56, *p* < 0.05), 72 h (F = 12.65, *p* < 0.05) and 96 h (F = 16.82, *p* < 0.05). Probit analysis revealed that the range of chronic (4 days) LC50 and LC90 values were 0.011 and 0.024 μL/insect, respectively (Table 5).

Extensive research has been conducted on the *Eucalyptus* genus for its effectiveness in controlling agricultural pests due to its insecticidal properties. We can list the work of Ainane et al. [50], they tested the *E. globulus* EO against insect pests of the flour (*T. confusum*), a concentration of 12 × 10^−2^ μL/cm^3^ caused the death of total mortality from the first day after treatment. Yang et al. [51] reported that *E. globulus* EO showed toxicity against human head lice, *Pediculus humanus* capitis. According to these authors, the insecticidal activity of *E. globulus* EO (LC50 = 0.125 mg/cm^2^) was higher than that of the commercial pediculides-delta-phenothrin (LC50 = 0.25 mg/cm^2^), and their mortality percentages were increased by increasing the EOs concentration and exposure time. Antitermitic tests demonstrated that *E. camaldulensis* EO from Thailand exhibited contact toxicity against *Coptotermes formosanus* (Isoptera: Rhinotermitidae), with LC50 values of 17.50 mg/g [52]. Ahouandjinou et al. [53] demonstrated that the EO of *E. camaldulensis* at a concentration of 2.5 µL.g^−1^ caused total mortality of *Sitophilus oryzae* (Coleoptera: Curculionidae), a major pest of rice, within 24 h of exposure.

In this study, spathulenol, cryptone, and o-cymene were identified as the main components of *E. camaldulensis* EO. These compounds are likely responsible for the oil’s potent insecticidal activity. According to Benelli et al. [54], spathulenol exhibited the strongest toxicity against *Metopolophium dirhodum* (Hemiptera: Aphididae), with LC50 equal to 4.3 mL/L. Also, it was reported that the *o*-cymene compound showed high contact toxicity against *Liposcelis bostrychophila* (Psocoptera: Liposcelididae), with an LC50 of 18.1 μg/adult [55].

#### 2.3.2. Fumigant Toxicity

Table 6 shows the percent mortality of *T. castaneum* after exposure to different concentrations of *E. camaldulensis* EO applied by fumigation method. These mortalities increase significantly according to the applied doses and the time after treatment of *T. castaneum*: at 24 h (F3.8 = 59.31; *p* < 0.005), 48 h (F3.8 = 20.75; *p* < 0.005) and 72 h (F3.8 = 17.33; *p* < 0.005) (Table 6).

The highest mortality in *T. castaneum* was achieved by the applied concentration of 666.67 μL/L Air (Mortality = 100%). The highest LC50 and LC90 values at 72 h exposure were 122.29 μL/L Air and 395.73 μL/L Air, respectively (Table 7).

The potential of EOs and their compounds as alternatives to currently used fumigants has been demonstrated in recent studies [56,57,58]. In the present study, it was demonstrated that *E. camaldulensis* EO possesses fumigant toxicity effects against the red flour beetle (*T. castaneum*). Several studies have confirmed the fumigant properties of *E. camaldulensis* EOs against stored product pests. Jemâa et al. [59] reported the fumigant toxicity of *E. camaldulensis* EO (LC50 = 18.96 μL/L) collected from Tunisia against *Ectomyelois cautella* (Lepidoptera: Pyralidae), a moth pest affecting stored dates. Similarly, Gad et al. [60] found high fumigant toxicity of *E. camaldulensis* EO from Egypt on eggs of *Tribolium confusum* (Coleoptera: Tenebrinoide), with an LC50 of 5.80 μL/L. According to Albouchi et al. [61], spathulenol, the main volatile component of *Melaleuca styphelioides*, was responsible for this tree’s essential oil fumigant toxicity against citrus aphid pest.

Using *E. camaldulensis* EO as a fumigant offers several advantages. It reduces the reliance on conventional chemical treatments, which often have drawbacks such as toxicity to non-target organisms, residue persistence, and potential human health risks. Furthermore, as pests like *T. castaneum* increasingly develop resistance to common fumigants [62], *E. camaldulensis* EO presents a promising, sustainable option due to its complex chemical profile that can be harder for pests to resist.

#### 2.3.3. Repellency Activity

Repellency activity of the *E. camaldulensis* EO against *T. castaneum* was given in Table 8. Our results revealed an increase in repellence effect of EO as function the exposure period and concentrations (Anova test; F = 638.95, *p* < 0.005). At concentrations of 0.8% and 0.6%, the EO exhibited strong repellent activity (class V) against *T. castaneum* with PR values of more than 85% at 30 min to 6 h post-exposure. At the lowest concentration (0.2%) *E. camaldulensis* EO showed moderate repellency activity (PR = 54.67%, Class III).

The toxicity results of tested EO against *T. confusum* adults revealed that the tested oils possessed potent repellent activity [63]. Similarly, Karemu et al. [64] noted the high repellent activity of *E. camaldulensis* EO (RP = 74.35%) from Kenya against *Sitophilus zeamais* (Coleoptera: Curculionidae), one of the most destructive pests of maize grains. According to Ali et al. [65], spathulenol isolated from *Tagetes patula* EO, showed high repellency against aphid pests. This compound exhibited a stronger repellent effect (PR = 70%) against the cigarette beetle *Lasioderma serricorne* (Coleoptera: Anobiidae) at 2 h after exposure, using a concentration of 1.57 nL/cm^2^ [66]. Also, it was reported that the spathulenol compound isolated from *Callicarpa* species demonstrated significant repellent activity against the yellow fever mosquito *Aedes aegypti* [67].

### 2.4. In Silico Investigations

**Figure 2:**
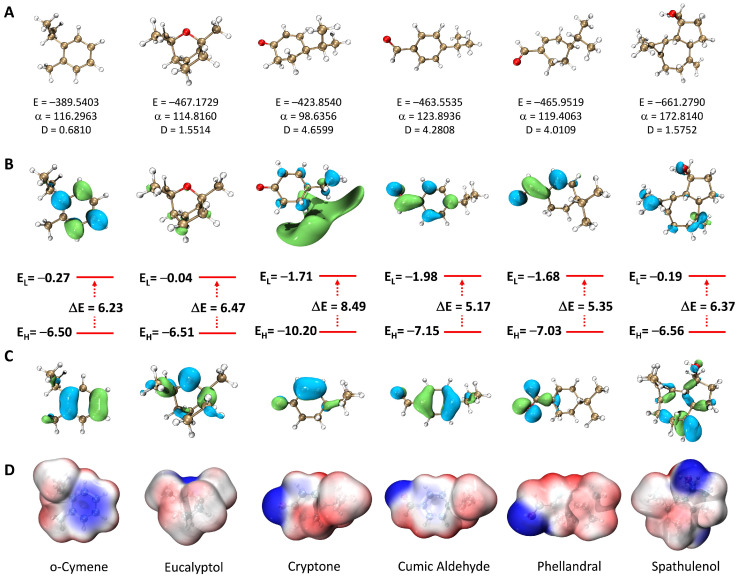
Molecular geometry (**A**), distribution and energies of LUMO (**B**) and HOMO (**C**), and ESP (**D**) of the *E. camaldulensis* essential oil compounds, calculated at the B3LYP/6-31+G(d,p) level.

To explore the correlation between the chemical composition of *E. camaldulensis* EO and its biological activity, computational studies were carried out on its main compounds. Firstly, the molecular structure and electronic properties of the six major compounds in the EO of *E. camaldulensis*, namely *o*-cymene, eucalyptol, cryptone, cuminaldehyde, phellandral, and spathulenol, were examined using quantum chemistry calculations. The DFT approach was chosen at the theoretical level B3 LYP/6-31+G(d,p). Three structural aspects were examined: molecular geometry, the distribution and energies of frontier molecular orbitals (FMOs), and electrostatic potential. The results obtained are presented in Figure 2. As can be seen, the six molecules examined possess an irregular spatial geometry due to the presence of methyl groups. The dipole moment varies between 0.6810 and 4.6599 Debye. The derivatives containing the carbonyl function, i.e., cryptone, cumic aldehyde, and phellandral, showed the highest values (4.0109–4.6599 Debye), followed by derivatives containing an oxygen atom eucalyptol and spathulenol (1.5514 and 1.5753 Debye), and finally, the hydrocarbon derivative *o*-cymene (0.6810 Debye). Furthermore, the polarizability of the molecules ranges from 98.6346 to 172.8140 a.u. The most polarized molecule is spathulenol, while the least polarized molecule is cryptone. Unlike the dipole moment, polarizability does not seem to be affected by the presence of the carbonyl group or the oxygen atom. Comparatively, the polarizability values of all the molecules are low, indicating that the molecules are relatively hard and unlikely to interact effectively with biological targets. Additionally, the electrostatic potential (ESP, Figure 2) shows that the oxygen atom is the richest site in electrons (blue region in the Figure) for all molecules except *o*-cymene. The richest electron site for the latter is the aromatic ring. Conversely, the positive charge (red region on the Figure) is distributed over several hydrogen atoms.

Furthermore, the HOMO (highest occupied molecular orbital) and LUMO (lowest unoccupied molecular orbital) of the molecules indicate that the FMOs of eucalyptol and spathulenol are sigma-type, while those of *o*-cymene, cryptone, cumic aldehyde and phellandral are pi-type. This distinction results from the presence of double bonds involved in resonance for the latter compounds.

The energy gaps (ΔE) of the molecules are within the range of 5.17 to 8.49 eV. Cryptone exhibited the highest value, while cumic aldehyde showed the lowest value. This indicates that the former is the least reactive molecule, while the latter is the most reactive molecule. The HOMO energies of the molecules vary between −6.50 and −10.20 eV. Cryptone showed the lowest value, indicating that it is the least effective electron donor. On the other hand, *o*-cymene and eucalyptol, which showed the highest HOMO energies (−6.50 and −6.51 eV), are the most potent electron donors. In comparison with reference antioxidants such as Trolox (E_HOMO_ = −5.39 eV) and BHT (E_HOMO_ = −5.74 eV), we can conclude that all the molecules are weak electron donors and thus are not good antioxidants [68,69,70].

After determining the molecular geometry of the major compounds of *E. camaldulensis* EO, we then examined their binding affinity towards three enzymes related to antimicrobial activity using molecular docking. The enzymes investigated are DNA gyrase, dihydrofolate reductase (DHFR), and Tyrosyl-tRNA synthetase (TyrRS).

DNA gyrase is an essential bacterial enzyme that catalyzes the ATP-dependent negative supercoiling of DNA. It is the only enzyme capable of actively introducing negative supercoils into DNA, a function crucial for maintaining the compact structure of bacterial genomes. Due to its importance, DNA gyrase is a prime target for several antibiotics. DHFR is an enzyme that catalyzes the conversion of dihydrofolate to tetrahydrofolate, an essential cofactor for DNA synthesis. DHFR is the target of the antibiotic trimethoprim, which inhibits this enzyme and thereby disrupts bacterial DNA replication. TyrRS is an enzyme that attaches the amino acid tyrosine to its corresponding tRNA molecule, an essential step in protein synthesis. Inhibition of this enzyme can disrupt bacterial translation and represent another potential target for antibiotics.

**Figure 3:**
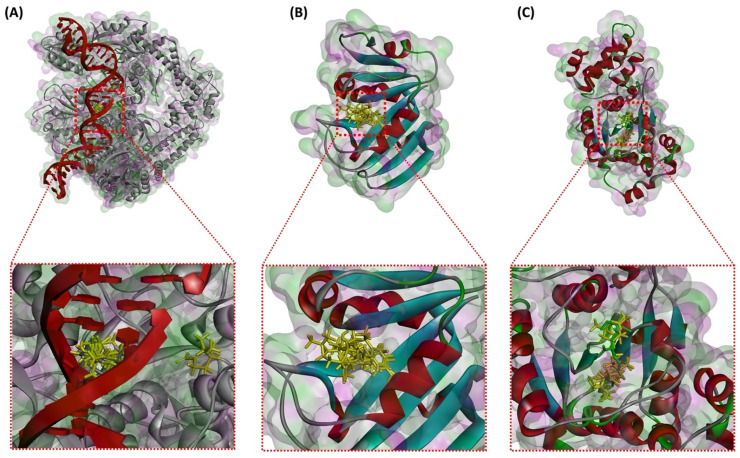
Superimposition of the docked models of *E. camaldulensis* EO compounds into the active site of DNA Gyrase (**A**), DHFR (**B**), and TyrRS enzymes (**C**).

**Figure 4:**
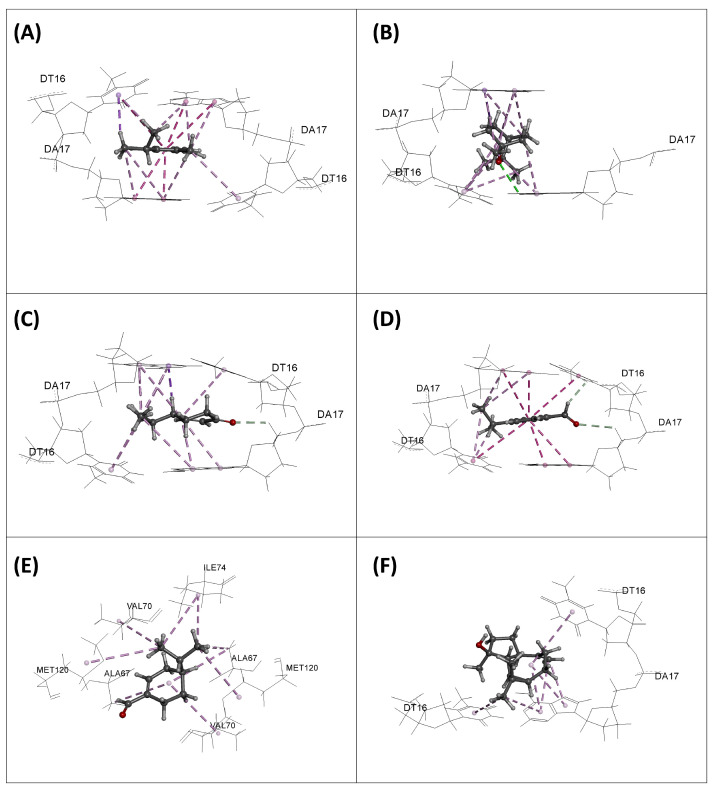
Interaction modes of *o*-cymene (**A**), eucalyptol (**B**), cryptone (**C**), cumic aldehyde (**D**), phellandral (**E**), and spathulenol (**F**) into the active site of DNA Gyrase enzyme.

**Figure 5:**
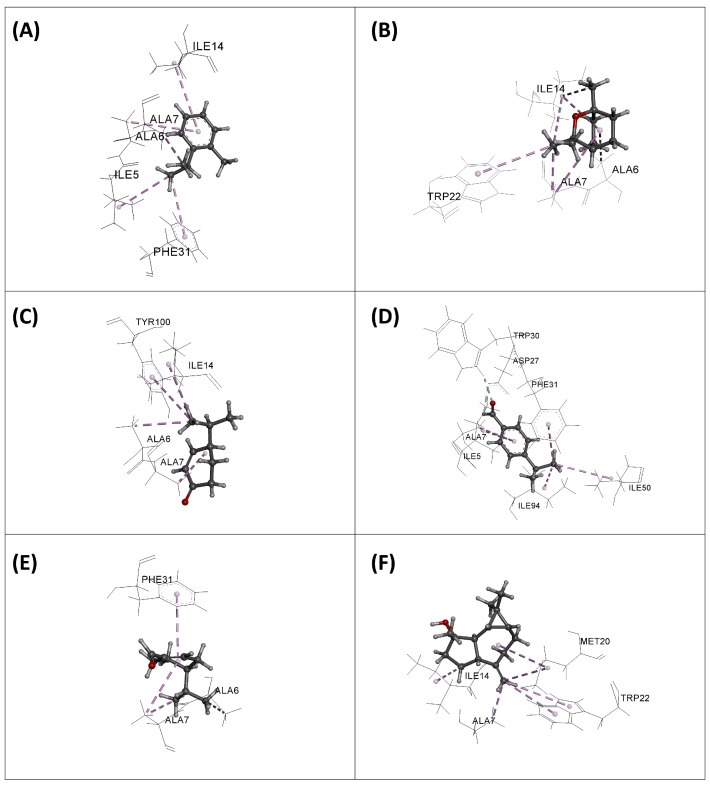
Interaction modes of *o*-cymene (**A**), eucalyptol (**B**), cryptone (**C**), cumic aldehyde (**D**), phellandral (**E**), and spathulenol (**F**) into the active site of DHFR enzyme.

**Figure 6:**
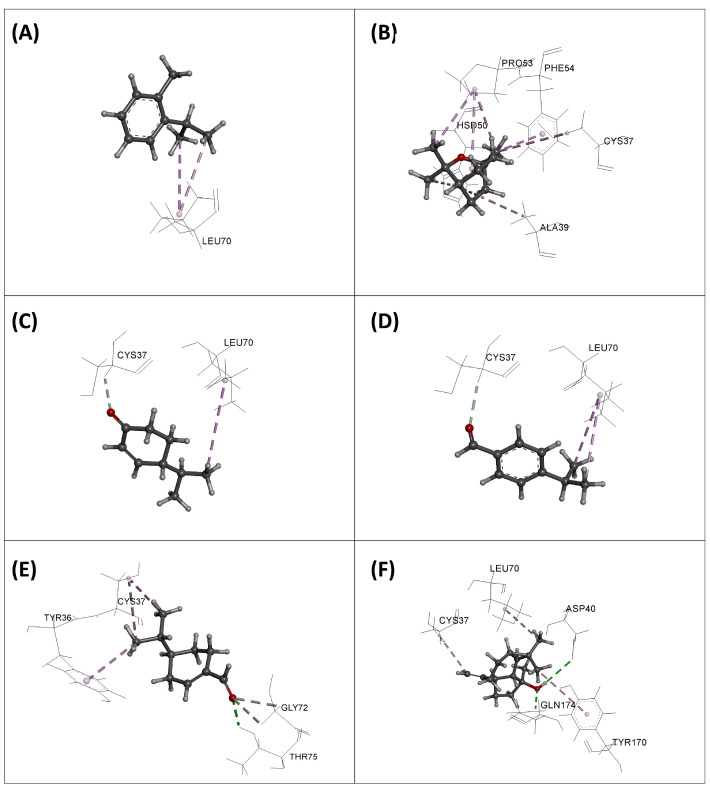
Interaction modes of *o*-cymene (**A**), eucalyptol (**B**), cryptone (**C**), cumic aldehyde (**D**), phellandral (**E**), and spathulenol (**F**) into the active site of TyrRS enzyme.

The results obtained are presented in Table 9 and Figure 3, Figure 4, Figure 5 and Figure 6. From the table, we observe that the molecules have a weak to moderate affinity towards the three enzymes studied. For DNA Gyrase, the binding affinities range from −2.04 to −3.39 kcal/mol. Compared to the standard gepotidacin (−5.98 kcal/mol), these values are relatively low, indicating that the molecules do not appear to be strong inhibitors of this enzyme. Similarly, and with comparable binding energies, none of the molecules show high affinity towards the DHFR and TyrRS enzymes, although they are slightly more active towards TyrRS than DHFR.

The superposition of the most stable orientations of the studied molecules in the active sites of the target enzymes is illustrated in Figure 3. Except for phellandral, which is relatively distant from the others in the case of DNA Gyrase, all the examined molecules overlap in the same region as the native ligands in the active sites of the enzymes. This indicates that, despite their low binding energy, their orientation in the active site could confer them an inhibitory activity against these enzymes.

The interaction modes of all the molecules are illustrated in Figure 4, Figure 5 and Figure 6. In the case of DNA Gyrase, we observe that all the molecules form favorable interactions exclusively with DNA, specifically the DNA fragments DT16 and DT17, except for phellandral, which forms contacts with the enzyme, notably with the amino acids VAL70, MET120, ALA67, and ILE74. These interactions are also present for the native ligand gepotidacin. Regarding the DHFR enzyme, the native ligand methotrexate mainly forms four hydrogen bonds with the residues ILE94, ARG57, ARG52, and LYS32. Additionally, it interacts with other amino acids through hydrophobic bonds, particularly PHE31, ILE5, ALA7, LEU28, and ILE50. Among the studied molecules, some can also form favorable interactions with these amino acids. For example, *o*-cymene forms hydrophobic interactions with PHE31, ALA7, and ILE5; eucalyptol, spathulenol, and cryptone form contacts with ALA7; cumic aldehyde interacts with PHE31, ALA7, ILE5, ILE94, and ILE50; and finally, phellandral forms interactions with PHE31 and ALA7. For the TyrRS enzyme, the reference ligand shows seven hydrogen bonds with CYS37, GLN196, GLY193, ASP195, TYR170, ASP80, and TYR36, and forms several hydrophobic bonds with LEU70, GLY38, PHE54, HSD50, and ALA39. In comparison with the studied molecules, we observe that they share some interactions with the native ligand. Overall, most molecules form favorable interactions with LEU70 and CYS37. Eucalyptol also forms interactions with HSD50, ALA39, and PHE54, while spathulenol forms interactions with LEU70 and TYR170.

The analysis of these results indicates that the molecules of *E. camaldulensis* EO are capable of binding to the active site of the three tested enzymes and form favorable interactions with key amino acids. However, their binding energy is relatively low, and the nature of the interactions with the enzyme is mainly of weak hydrophobic type. This suggests that these molecules may exhibit inhibitory activity towards the enzymes DNA Gyrase, DHFR, and TyrRS, but with relatively low capacity. Moreover, the possibility that these compounds may interact with other biological targets warrants further investigation to better understand the full scope of their antimicrobial effects.

## 3. Conclusions

This study highlights the potent antimicrobial and insecticidal properties of *E. camaldulensis* essential oil, which showed strong inhibitory effects against various bacterial and fungal strains, particularly *S. typhimurium*, *S. aureus*, *C. albicans*, and *Penicillium* sp. Additionally, the oil demonstrated notable insecticidal activity against *T. castaneum*, underscoring its potential for use in pest control applications. Furthermore, molecular docking studies revealed that the major constituents of the EO exhibit moderate affinity for key enzymes such as DNA gyrase, DHFR, and TyrRS. However, their most stable mode of interaction occurs at the active site of these enzymes, which could partly explain their antimicrobial activity. Future studies are recommended to determine Minimum Inhibitory Concentration (MIC) values for individual components to allow for a more precise assessment of their antimicrobial efficacy.

## 4. Materials and Methods

### 4.1. Plant Material and Essential Oil Extraction

Adult fresh of *E. camaldulensis* leaves were collected in March 2023 from one adult tree in Oued-Al-Athmania area at Mila province, Northeast of Algeria (latitude 36°14′59″ N and longitude 6°17′10″ E). The harvested plant material was air-dried for two weeks and then ground using a small laboratory mill. The essential oil was extracted from 125 g of powdered leaves using a Clevenger-type apparatus for hydro-distillation at 100 °C for 75 min [71]. The resulting oil was kept in a refrigerator at 4 °C in an amber-colored glass bottle until it was ready for use in GC–MS characterization and lab experiments [72].

To calculate the oil yield of dried *E. camaldulensis* leaves, the formula yield (%) was used. This formula takes into account the dry essential oil volume and the weight of the dried *E. camaldulensis* leaves.

(1) $$Yield\left(\%\right)=\frac{weight\;of\;collected\;extract}{dry\;weight\;of\;the\;extracted\;sample}\times 100$$

### 4.2. GC-MS Analysis

GC-MS measurements were performed with an Agilent 6850 GC instrument (Milan, Italy) coupled to an Agilent 5973 Inert MS. 1 µL of the sample was injected with a split ratio 1:20 into an HP-5ms Ultra inert GC capillary column (stationary phase: (5%-phenyl)-methylpolysiloxane; Length: 30 m; ID: 0.25 mm; Film Thickness: 0.25 µm). The injection temperature was 250 °C, and the temperature ramp raised the column temperature from 70 °C to 280 °C: 70 °C for 1 min, 10 °C/min until the column temperature reached 170 °C, and 30 °C/min until the column temperature reached. 280 °C. Subsequently, it was held at 280 °C for 5 min. Helium was used as a carrier gas at a flow rate of 1 mL/min. The solvent delay was set to 4 min. Measurements were performed under electron impact (EI) ionization (70 eV) in full scan mode (*m*/*z* 35–550) at a frequency of 3.9 Hz. The EI ion source and quadrupole mass filter temperatures were kept, respectively, at 200 and 250 °C. Compounds were identified by comparing their EI mass spectra at 70 eV with spectra of known substances collect in the NIST20 mass spectral library (NIST 20, https://www.nist.gov/srd/nist-standard-reference-database-1a, accessed on 10 October 2024). Moreover, the identification was supported by Kovats retention index (RI) calculated for each analyte by the Kovats equation, using the standard n-alkane mixture in the range C7-C40 (Sigma-Aldrich, Saint Louis, MO, USA).

The relative percentage of each compound is defined by the area of selected peak divided for the sum of the peak area.

### 4.3. Antibacterial Activity Test

The antibacterial effects of the *E. camaldulensis* EO was evaluated using disc diffusion technique against six bacterial strains: two Gram-positive (*Staphylococcus aureus* (ATCC 25923), *Bacillus subtilis* (ATCC6633)) and four Gram-negative (*Escherichia coli* (ATCC 25922), *Pseudomonas aeruginosa* (ATCC 27853), *Salmonella typhimurium* (ATCC 14028) and *Klebsiella pneumoniae* (ATCC 700603)). Bacterial strains were reactivated and incubated at 37 °C for 24 h and then emulsified in physiological water to obtain a cell suspension turbidity matches 0.5 Mc Farland corresponding to 10^6^ CFU/mL [73]. Sterile paper discs of 6 mm prepared from Wattman paper No. 1 impregnated with 10 µL of sample (1 mg EO was dissolved in 10 µL DMSO) were dispensed onto the surface of the inoculated Mueller-Hinton plate. The dishes were left for 2 h at 4 °C to allow the diffusion of the bioactive substances and then they were incubated at 37 °C. Each test was realized in triplicates. Antibacterial results’ reading was done after 18 to 24 h. Any growth inhibition zone around discs, even of a small diameter, was considered a positive result. Antibacterial agent gentamicin (10 µg) was used as a positive control in order to compare their antibacterial potency with our essential oil, and DMSO was used as a negative control [74].

### 4.4. Antifungal Activity Test

Antifungal activity of the same concentration of *E. camaldulensis* EO was tested by the same disc technique described previously against four fungal strains: *Aspergillus niger* (MH109542), *Aspergillus fumigatus* (MH109539), *Candida albicans*, *Penicillium* sp. The fungi were cultured on Potato Dextrose Agar (PDA) medium and then incubated at 28 °C for a period of 14 days. Dense suspensions containing spores and mycelial fragments were obtained by scraping the cultures after adding physiological water. These suspensions were then diluted until they reached an absorbance of 0.15–0.20 at 650 nm. This suspension is diluted to 1/10th [75]. However, for *Candida albicans* the suspension was adjusted to a turbidity equivalent to the 0.5 McFarland standard [76]. The test was carried out on Sabouraud medium, and the dishes were incubated at 28 °C. Antifungal results reading was done after 48 and 72 h. Parallel analysis study with a commercial antimicrobial agent nystatin (1 mg/mL) was conducted in order to compare their antifungal potency with our essential oil. DMSO was used as a negative control [77,78].

### 4.5. Insecticidal Activity

#### 4.5.1. Test Insect Culture

Red flour beetles *Tribolium castaneum* (Coleoptera: Tenebrionidae) was selected for this study because it is a major pest of stored cereal products, causing significant economic losses worldwide [79]. Its high reproductive rate, adaptability to various storage conditions, and resistance to conventional chemical treatments make it a persistent threat in storage facilities [80]. Studying this pest is crucial for developing effective, sustainable pest management strategies that protect cereal quality and reduce post-harvest losses.

*Tribolium castaneum* used in this study were obtained from cultures stored in the laboratory using wheat flour. A plastic bottle measuring 25 cm in height and 12 cm in width was loaded with wheat flour (200 g) containing brewer’s yeast 5% (*w*/*w*). The adult insects that were 4 mm × 1.5 mm in size were moved to new bottles after 5 days of oviposition, and to obtain uniform age adults, the jars were regularly checked. The *T. castaneum* cultures were maintained at 28 ± 2 °C and 80 ± 5% relative humidity. Two weeks old *T. castaneum* adults were used in all insect bioassays. All tests were carried out under conditions identical to those of the cultures. In all bioassays, insects were considered dead when no leg or antennal movements were observed.

#### 4.5.2. Contact Toxicity

The test was carried out according to the method described by Pinto et al. [81] and Saada et al. [82]. Several preliminary tests were carried out to select the doses to be used for *E. camaldulensis* EO. Five concentrations (1, 1.5, 2, 2.5, and 3%) were prepared by diluting 10, 15, 20, 25, and 30 µL of EO in 1 mL acetone providing corresponding concentrations of 0.01, 0.015, 0.02, 0.25 and 0.03 µL/insect. Practically, 1 μL of each concentration was directly applied to the thorax of the insect. Controls were determined using Acetone. Ten insects were used for each concentration and control, and the experiment was replicated three times. Both treated and control insects were then transferred to the Petri dishes (90 mm diameter) containing 10 g of crushed sterile wheat seeds. To avoid fumigant toxicity, a plastic lid perforated on top lid was used to cover the Petri plates. Mortality was observed after 24, 48, 72 and 96 h of treatment application. Contact toxicity concentration was expressed as μL of EO required to give toxic effect per adult. The percentage of insect mortality was calculated using the Abbott correction formula for natural mortality in untreated controls [83] and the concentrations that cause 50% mortality (LC50) or 90% mortality (LC90) as well as their confidence limits at 95% were also worked out.

(2) $$Corrected\;mortality\;\left(\%\right)=\left(1-\frac{n\;in\;T\;after\;treatment}{n\;in\;Co\;after\;treatment}\right)\times 100$$

where *n* = number of insects, *T* = treated and *Co* = control.

#### 4.5.3. Fumigant Toxicity

Fumigant toxicity of *E. camaldulensis* EO on the longevity of *T. castaneum* adults was evaluated by the method described previously [84]. The experiments were done in 60 mL Falcon tube (5 × 4 cm) with screw caps. Cotton pieces were fixed to the inner side of the caps with cotton threads and impregnated with 10, 20, 30, and 40 μL of EO, corresponding to respective calculated concentrations of 166.67, 333.33, 500, and 666.67 μL/L air. Ten adults of the test insect were transferred into the plastic bottle, which was tightly closed. For each dose, three replicates were performed. The comparison was made with a control sample (cotton without test solutions). After 24 h, 48 h and 72 h, the number of dead insects was recorded, and the LC50 and LC90 values were calculated with the help of Abbott’s equation as described in Section 4.5.2. The result was expressed as μL EO/L volume required to kill the insects.

#### 4.5.4. Repellent Activity

The repellent effect of *E. camaldulensis* EO against the adults of *T. castaneum* was assessed using the method of Mc Donald et al. [85]. In 500 μL of acetone, the following doses of *E. camaldulensis* were diluted: 2, 4, 6, and 8 μL, which were equivalent to 0.06, 0.12, 0.18 and 0.26 mg/cm^2^, respectively. A disc of Whatman paper (N°1) with a 9 cm diameter was cut into two halves. The first half was treated with 500 μL of the prepared sample, while the same volume of acetone was used to treat the second half. After allowing the Whatman paper and control to dry for 10 min, they were adhered together with adhesive and placed inside Petri dishes. Ten adults were introduced into the middle of each filter paper disc, and the number of insects present on each half was counted after 30 min, 1, 2, 4 and 6 h. Each treatment was replicated three times. The repulsion percentage (*RP*) was calculated according to McDonald formula [85]:

(3) $$RP=\frac{\left(NC-NT\right)}{\left(NC+NT\right)}\times 100$$

where *RP* represents the percentage of repellence, *NC* represents the number of test insects in the control half disc, and *NT* represents the number of test insects in the treated half-disc.

The results were classified into different classes based on the percentage of repellence observed, ranging from 0% to 100% [85]. The following classes were used to group the percentage repellency obtained: class 0 (0 to 0.1%), class I (0.2 to 10%), class II (10.1 to 40%), class III (40.1 to 60%), class IV (60.1 to 80%), and class V (80.1 to 100%) [68].

### 4.6. DFT Calculations

The molecular geometry and electronic properties of the main compounds of *E. camaldulensis* EO were studied using density functional theory (DFT) calculations. These calculations were performed using Gaussian 09 software [86], using the B3LYP function in conjunction with the 6-31+G(d,p) basis set. Validation of the fundamental states was ensured by checking the absence of imaginary frequencies. The analysis and visualization of the results have been performed using Multiwfn 3.8 and VMD 1.9.3 software [87,88].

### 4.7. Molecular Docking

The molecular geometry of the molecules studied was obtained from DFT calculations. The coordinates of the E. coli DNA gyrase enzyme (PDB code: 6RKU), E. coli dihydrofolate reductase enzyme (DHFR, PDB code: 4DFR), and S. aureus Tyrosyl-tRNA synthetase enzyme (TyrRS, PDB code: 1JIJ) were obtained from the Protein Data Bank. Ligands, water molecules, heteroatoms, and co-crystallised solvents were removed from the proteins, and partial charges and hydrogens were added to the protein using LePro software (http://www.lephar.com, accessed on 1 September 2024). The docking search space was defined as a 25 Å cube with grid points 1 Å apart, centred on the active site of the protein (DNA gyrase: x = 158.1506; y = 158.7073; z = 146.7502; DHFR: x = 18.4407; y = 68.7857; z = 42.6854; TyrRS: x = −11.4226; y = 14.9726; z = 85.9476). LeDock (http://www.lephar.com, accessed on 1 September 2024) software was used to perform molecular docking studies. Figures were created using BIOVIA Discovery Studio 2019. The docking protocol was validated by comparing crystallographic and theoretical data for the native ligands, giving an RMSD value lower than 2 Å.

### 4.8. Statistical Analysis

All measurements were taken three times for each treatment (antibacterial, antifungal and insecticidal). The results were analyzed by one-way analysis of variance (ANOVA) followed by Tukey HSD post hoc test for multiple comparisons. Differences were considered significant at *p* < 0.05. Insect mortality data recorded were also subjected to probit analysis for calculating LC 50 and LC 90 values. All the analyses were performed by using SPSS software version 25.0.

## Figures and Tables

**Table 1:** Phytochemical constituents contained in the EO extracted from Algerian *E. camaldulensis.* MF represents the Match Factor calculated by comparing the experimental mass spectrum with those reported in NIST20 mass spectral library, MF = 1000 corresponds to a perfect match between a pair of compared spectra RI_exp_ and RI_NIST20_ represent, respectively, Kovats programmed temperature retention indices evaluated in this work (on a 5% phenylmethylpolysiloxane) and gathered from NIST20 mass spectral library (for identical or similar) stationary phases. %ΔRI is the percent difference between RI_exp_ and RI_NIST20_.

No.	Compounds	MF	RI_exp_	RI_NIST20_	%ΔRI	Peak Area (%)
1	*o*-Cymene	839	1022	1027	−0.49	15.53
2	Eucalyptol	923	1034	1032	0.19	1.22
3	Cryptone	904	1226	1192	2.85	17.22
4	Cumic aldehyde	941	1273	1279	−0.47	2.92
5	Phellandral	865	1304	1281	1.80	3.63
6	Carvacrol	908	1323	1328	−0.38	0.81
7	Aromandendrene	920	1486	1455	2.13	0.44
8	Spathulenol	931	1616	1620	−0.25	58.24

**Table 2:** Antibacterial activity of essential oils from *E. camaldulensis* compared to an antibiotic.

	Diameter of Inhibition Zone in mm					
	*S. aureus*	*B. subtilis*	*E. coli*	*P. aeruginosa*	*S. typhimurium*	*K. pneumoniae*
EO	14.5 ± 0.50 ^c^	11 ± 1.00 ^d^	10 ± 0.41 ^d^	-	14 ± 1.00 ^c^	8 ± 0.30 ^e^
Gentamicin	18 ± 0.51 ^ab^	19 ± 0.61 ^a^	11 ± 0.32 ^d^	17 ± 0.25 ^b^	15 ± 0.37 ^c^	10 ± 0.55 ^d^

(-): No activity; Superscript values with different letters within the same row are significantly different (*p* < 0.05).

**Table 3:** In vitro analysis of the antifungal activity of *E. camaldulensis* EO with disc diffusion method.

	Diameter of Inhibition Zone in mm			
	*A. niger*	*A. fumigatus*	*Penicillium* sp.	*C. albicans*
EO	7.0 ± 0.50 ^g^	9.8 ± 0.27 ^f^	11.5 ± 0.49 ^e^	11.2 ± 0.29 ^e^
Nystatin	18.8 ± 0.28 ^b^	25.5 ± 0.52 ^d^	28.5 ± 0.51 ^a^	21.5 ± 0.48 ^c^

Superscript values with different letters within the same row are significantly different (*p* < 0.05).

**Table 4:** Corrected percent mortality (means ± SE) of *T. castaneum* adults after application of different concentrations of *E. camaldulensis* EO at different time intervals.

Concentration (μL/Insect)		Mortality (%)			
		24 h	48 h	72 h	96 h
0.010		30 ± 10.0 ^c^	33.33 ± 8.82 ^c^	33.33 ± 8.82 ^b^	36.67 ± 8.22 ^c^
0.015		63.33 ± 6.66 ^b^	63.33 ± 6.66 ^b^	66.67 ± 8.82 ^a^	70.0 ± 10.0 ^b^
0.200		66.67 ± 3.33 ^b^	66.67 ± 3.03 ^b^	73.33 ± 6.67 ^a^	80.0 ± 2.0 ^ab^
0.250		76.67 ± 3.33 ^ab^	76.66 ± 3.31 ^ab^	83.33 ± 6.67 ^a^	86.67 ± 3.33 ^ab^
0.300		96.67 ± 3.32 ^a^	96.67 ± 3.32 ^a^	100.0 ^a^	100.0 ^a^
One-Way-Anova	F value	16.56	16.64	12.65	16.82
	*p* value	<0.005	<0.005	<0.005	<0.005

Means within the same column followed by same letter are not significantly different (*p* < 0.05).

**Table 5:** Lethal concentrations on contact activity of *E. camaldulensis* essential oils against *T. castaneum* at different exposure times.

Exposure Time (h)	LC_50_ ^a^ (μL/Insect)	LC_90_ ^a^ (μL/Insect)	Slope ± SEM ^b^	Chi-Square (χ^2^)	df
24	0.014 (0.008–0.017)	0.03 (0.023–0.086)	6.96 ± 2.16	1.3	3
48	0.013 (0.007–0.017)	0.031 (0.023–0.104)	6.64 (2.14)	1.28	3
72	0.013 (0.006–0.016)	0.026 (0.020–0.055)	7.93 (2.31)	1.29	3
96	0.011 (0.006–0.015)	0.024 (0.019–0.047)	8.27 (2.40)	0.9	3

LC: lethal concentration; ^a^ Values in the bracket represent lower and upper confidence limit; ^b^ SEM: Mean standard error.

**Table 6:** Percent mortality of *T. castaneum* after exposure to different concentrations of *E. camaldulensis* EO applied by fumigation method.

Concentration		24 h	48 h	72 h
166.67 μL/L Air		10 ± 1.10 ^c^	40.0 ± 6.67 ^c^	65 ± 2.88 ^b^
333.33 μL/L Air		50 ± 10.0 ^b^	76.66 ± 6.66 ^b^	85.0 ± 2.89 ^a^
500 μL/L Air		85 ± 2.88 ^a^	8.05 ± 2.74 ^ab^	90 ±5.77 ^a^
666.67 μL/L Air		100.0 ^a^	100.0 ^a^	100.0 ^a^
One-Way-Anova	F value	59.31	20.75	17.33
	*p* value	<0.005	<0.005	<0.005

Means within the same column followed by same letter are not significantly different (*p* < 0.05).

**Table 7:** Lethal concentrations of *E. camaldulensis* EO against *T. castaneum* adults at different exposure times.

Exposure Time (h)	LC_50_ ^a^ (µL/Liter Air)	LC_90_ ^a^ (µL/Liter Air)	Slope ± SEM ^b^	Chi-Square (χ^2^)	df
24	294.02 (405.07–600.27)	724.60 (597.37–1370.24)	−20.75 ± 6.44	0.021	2
48	203.15 (82.01–282.24)	485.206 (345.95–1369.53)	−7.82 ± 2.80	0.822	2
72	122.294 (0.003–212.25)	395.73 (239.24–27,233.56)	−2.25 ± 2.93	0.605	2

LC: lethal concentration; ^a^ Values in the bracket represent lower and upper confidence limit; ^b^ SEM: Mean standard error.

**Table 8:** Repellent activity (%) of *E. camaldulensis* EO against *T. castaneum* at different exposure times and concentrations.

Concentration		Repellence Per Cent of Treatments After					Mean Repellence	Repellent
		30 min	1 h	2 h	4 h	6 h		Class
0.2% (0.06 mg/cm^2^)		40.0 ± 0.58 ^c^	40.0 ± 0.58 ^c^	53.34 ± 1.86 ^d^	60 ± 1.0 ^d^	80 ± 0.58 ^d^	54.67 ± 7.42 ^c^	III
0.4% (0.12 mg/cm^2^)		73.33 ± 0.33 ^b^	80.0 ± 0.58 ^a^	73.34 ± 0.33 ^c^	66.67 ± 0.66 ^c^	86.66 ± 0.33 ^c^	76.0 ± 3.40 ^b^	IV
0.6% (0.18 mg/cm^2^)		73.34 ± 0.33 ^b^	66.66 ± 0.88 ^b^	100 ^b^	86.67 ± 0.33 ^a^	100 ^a^	85.33 ± 6.80 ^b^	V
0.8% (0.26 mg/cm^2^)		80.0 ± 0.58 ^a^	80 ± 0.41 ^a^	93.33 ± 0.33 ^a^	75.33 ± 0.67 ^b^	100 ^a^	85.73 ± 4.66 ^a^	V
One-Way-Anova	F value	280.01	922.07	286.33	110.76		638.95	
	*p* value	<0.005	<0.005	<0.005	<0.005		<0.005	

Means within the same column followed by same letter are not significantly different (*p* < 0.05).

**Table 9:** Docking binding energies in kcal/mol of *E. camaldulensis* EO compounds with DNA Gyrase (6rku), DHFR (4dfr), and TyrRS (1jij) enzymes.

Compound	Docking Binding Energy in kcal/mol		
	DNA Gyrase	DHFR	TyrRS
*o*-Cymene	−3.07	−1.93	−3.30
Eucalyptol	−2.04	−1.96	−2.50
Cryptone	−3.39	−2.07	−3.21
Cumic aldehyde	−3.22	−2.40	−3.75
Phellandral	−2.66	−2.52	−3.28
Spathulenol	−3.13	−2.43	−3.53
Native ligand	−5.98	−7.35	−5.75

## Data Availability

The data presented in this study are available on request from the corresponding author.
